# The autophagy protein ATG9A promotes HIV-1 infectivity

**DOI:** 10.1186/s12977-019-0480-3

**Published:** 2019-07-03

**Authors:** Elodie Mailler, Abdul A. Waheed, Sang-Yoon Park, David C. Gershlick, Eric O. Freed, Juan S. Bonifacino

**Affiliations:** 1 Cell Biology and Neurobiology Branch, Eunice Kennedy Shriver National Institute of Child Health and Human Development, National Institutes of Health, Bethesda, MD 20892 USA; 2 HIV Dynamics and Replication Program, National Cancer Institute, National Institutes of Health, Frederick, MD 21702 USA; 3 Present Address: Cambridge Institute for Medical Research, University of Cambridge, Cambridge, UK

**Keywords:** Nef, ATG9A, Autophagy, Virus release, Infectivity

## Abstract

**Background:**

Nef is a multifunctional accessory protein encoded by HIV-1, HIV-2 and SIV that plays critical roles in viral pathogenesis, contributing to viral replication, assembly, budding, infectivity and immune evasion, through engagement of various host cell pathways.

**Results:**

To gain a better understanding of the role of host proteins in the functions of Nef, we carried out tandem affinity purification-mass spectrometry analysis, and identified over 70 HIV-1 Nef-interacting proteins, including the autophagy-related 9A (ATG9A) protein. ATG9A is a transmembrane component of the machinery for autophagy, a catabolic process in which cytoplasmic components are degraded in lysosomal compartments. Pulldown experiments demonstrated that ATG9A interacts with Nef from not only HIV-1 and but also SIV (cpz, smm and mac). However, expression of HIV-1 Nef had no effect on the levels and localization of ATG9A, and on autophagy, in the host cells. To investigate a possible role for ATG9A in virus replication, we knocked out ATG9A in HeLa cervical carcinoma and Jurkat T cells, and analyzed virus release and infectivity. We observed that ATG9A knockout (KO) had no effect on the release of wild-type (WT) or Nef-defective HIV-1 in these cells. However, the infectivity of WT virus produced from ATG9A-KO HeLa and Jurkat cells was reduced by ~ fourfold and eightfold, respectively, relative to virus produced from WT cells. This reduction in infectivity was independent of the interaction of Nef with ATG9A, and was not due to reduced incorporation of the viral envelope (Env) glycoprotein into the virus. The loss of HIV-1 infectivity was rescued by pseudotyping HIV-1 virions with the vesicular stomatitis virus G glycoprotein.

**Conclusions:**

These studies indicate that ATG9A promotes HIV-1 infectivity in an Env-dependent manner. The interaction of Nef with ATG9A, however, is not required for Nef to enhance HIV-1 infectivity. We speculate that ATG9A could promote infectivity by participating in either the removal of a factor that inhibits infectivity or the incorporation of a factor that enhances infectivity of the viral particles. These studies thus identify a novel host cell factor implicated in HIV-1 infectivity, which may be amenable to pharmacologic manipulation for treatment of HIV-1 infection.

**Electronic supplementary material:**

The online version of this article (10.1186/s12977-019-0480-3) contains supplementary material, which is available to authorized users.

## Background

**Fig. 1:**
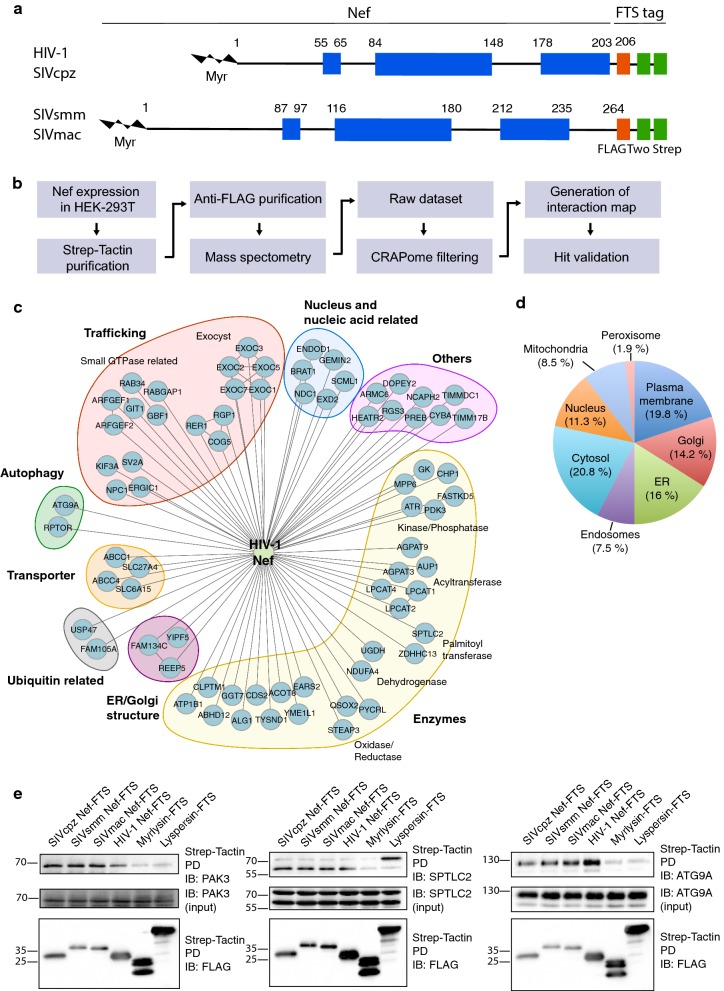
Identification and validation of HIV-1 and SIV Nef interactors. **a** Schematic representation of FTS-tagged Nef constructs used for TAP-MS. Nef is myristoylated (Myr) at the N-terminus and comprises three folded domains (represented in blue) (residues 55–65, 84–148 and 178–203 in HIV-1 Nef; residues 87–97, 116–180 and 212–235 in SIVsmm Nef) flanked by flexible segments, comprising an N-terminal flexible anchor (residues 1–54 in HIV-1 Nef; residues 1–86 in SIVsmm Nef) and a C-terminal flexible loop (residues 149–177 in HIV-1 Nef; residues 181–211 in SIVsmm Nef). SIVcpz Nef is similar to HIV-1 Nef and SIVmac to SIVsmm. The FTS tag is composed of one FLAG tag (DYKDDDK) followed by two strep tags (WSHPQFEK). **b** Flowchart of the TAP-MS protocol conducted to identify Nef-interacting proteins. Detergent extracts of HEK-293T cells stably expressing FTS-tagged Nef from the HIV-1 NL4-3 strain or from three SIV strains (SIVcpz, SIVmac and SIVsmm) were incubated with Strep-Tactin beads and bound proteins eluted with desthiobiotin. The eluate was further purified by binding to anti-FLAG M2 beads and elution with FLAG peptide. Eluted proteins were identified by MS. Raw data were filtered against the CRAPome database, and interaction maps were generated using BioGRID. **c** Interaction map of HIV-1 Nef with host proteins identified by TAP-MS and grouped according to their cellular functions. **d** Classification of HIV-1 Nef interactors according to their cellular compartments. **e** Validation of TAP-MS hits by pulldown (PD) and immunoblotting (IB). HeLa cells were transfected with plasmids encoding Nef from HIV-1 or the three SIV strains, as well as myrlysin or lyspersin as specificity controls, all tagged with FTS. Cells were cross-linked, lysed and the FTS-tagged proteins isolated on Strep-Tactin beads. The isolated proteins were analyzed by SDS-PAGE and immunoblotting with antibodies to endogenous PAK3, SPTLC2 and ATG9A, as well as antibody to the FLAG epitope. The positions of molecular mass markers (in kDa) are indicated on the left. The > 70-kDa species observed in the lyspersin-FTS pulldown is likely a non-specific protein that is recognized by the antibody to SPTLC2

Viruses need to circumvent the intrinsic defenses of their hosts in order to replicate and disseminate. To this end, primate lentiviruses such as human immunodeficiency viruses (HIV-1, HIV-2) and simian immunodeficiency viruses (SIV) have evolved to encode several virulence factors that create favorable conditions for viral replication within their host cells. Prominent among these factors is Nef, an accessory protein encoded in all HIV-1, HIV-2 and SIV genomes that is highly expressed early after infection (reviewed in Refs. [1, 2]). Nef is a 27–35 kDa N-terminally myristoylated protein that associates with the cytosolic face of membranes (Fig. 1a). Structural studies have shown that Nef comprises several folded segments (comprising residues 55–65, 84–148 and 178–203) flanked by flexible segments [3–6] (Fig. 1a) (amino-acid residue numbers correspond to the NL4-3 variant of HIV-1 Nef). A flexible loop connecting the folded 84–148 and 178–203 segments becomes structured when it binds to one of its targets, the clathrin-associated adaptor protein complex AP-2 [7].

Nef has been reported to promote multiple aspects of the viral life cycle, including replication, assembly, budding, infectivity and immune evasion, through engagement of various cellular pathways [1, 2]. Among these pathways are protein trafficking, intracellular signaling and autophagy. In some cases, Nef causes redistribution and/or degradation of cellular proteins [8–10], as exemplified by the downregulation of the HIV-1 receptor CD4 [11] and coreceptors CCR5/CXCR4 [12], class I molecules of the histocompatibility complex (MHC-I) [13] and the viral restriction factors SERINC3 and SERINC5 [14–18], from the surface of infected cells. The mechanisms of Nef-induced CD4 and MHC-I downregulation are well understood, involving the formation of tripartite complexes of these proteins with Nef and the clathrin adaptors AP-2 [7] and AP-1 [6], respectively. Nef is also capable of activating various cellular kinases such as members of the p21-activated kinase (PAK) [19, 20] and Src families [21]. Furthermore, Nef has been reported to inhibit autophagy through interactions with components of the autophagy machinery such as beclin 1 [22, 23], syntaxin 17 and IRGM [24]. However, many aspects of Nef function remain insufficiently understood, so there is a need to identify additional host cell targets. Several methods have been used to identify HIV-1 Nef interactors, including classical yeast two-hybrid screens [25, 26], a split-ubiquitin-based yeast two-hybrid system [27], affinity purification followed by peptide microsequence analysis [28] and proteomic analysis using tandem affinity purification (TAP)-mass spectrometry (MS) [29–32]. Although a number of Nef-binding proteins were identified in these studies, many have not been independently validated and their potential roles in Nef function and HIV pathogenesis remain to be determined.

To undertake a more comprehensive and detailed analysis of lentiviral Nef interactors, we performed TAP-MS using as baits the Nef proteins from one HIV-1 strain (NL4-3) and three SIV strains [from chimpanzee (cpz), macaque (mac) and sooty mangabey (smm)] expressed by stable transfection into HEK-293T cells. After filtering the results to remove frequent contaminants, we ended up with dozens of proteins that interacted with one or more of the Nefs. Among these, we chose for further study the transmembrane autophagy protein ATG9A. We found that Nef and ATG9A did not alter each other’s localization or levels in HeLa cervical carcinoma or Jurkat T cells. Moreover, expression of Nef had little or no effect on autophagy in these cells. However, while knock-out (KO) of the *ATG9A* gene did not affect the release of HIV-1 particles, it greatly decreased the infectivity of the particles. This effect was independent of the interaction of ATG9A with Nef, but dependent on the envelope glycoprotein (Env) of HIV-1. These findings thus identified ATG9A as a novel host cell factor that promotes the production of infectious HIV-1 particles in a Nef-independent but Env-dependent manner.

## Results

### Identification of host cell proteins that interact with HIV-1 and SIV Nef

To identify host cell proteins that interact with Nef, we conducted a TAP-MS analysis, following the procedure outlined in Fig. 1b. Nef proteins from the HIV-1 NL4-3 strain and the three SIV strains (cpz GAB1, mac239 and smm FWR1) were tagged with sequences encoding one FLAG and two strep tags (herein referred to as FLAG-Two-Strep or FTS) at the C-terminus (Fig. 1a), and expressed by stable transfection in HEK-293T cells. Detergent extracts from these cells were subjected to sequential affinity purification on Strep-Tactin and anti-FLAG M2 affinity beads. Affinity-purified proteins were identified by liquid chromatography followed by MS (Additional file 1: Table S1). The results were filtered against the Contaminant Repository for Affinity Purification Mass Spectrometry Data (CRAPome) database [33] to remove frequent contaminants in TAP-MS. This procedure yielded 73 proteins that co-purified with HIV-1 Nef (Fig. 1c), 17 with SIVcpz Nef, 27 with SIVmac Nef and 19 with SIVsmm Nef (Additional file 2: Figure S1A). The interactors included proteins that are involved in trafficking, autophagy, solute transport, ubiquitination, ER/Golgi structure, nuclear processes and cellular metabolism (Fig. 1c), and that localize to various cellular compartments (Fig. 1d). Some interactors were common to two or more Nefs. Among these were the subunits of the exocyst (i.e., EXOC1-EXOC8) (Fig. 1c and Additional file 2: Figure S1A, B), a tethering complex that was previously shown to interact with HIV-1 Nef, and to promote Nef-mediated enhancement of nanotube formation [30] and regulation of actin remodeling [34]. Other common interactors included components of a complex comprising the p21 activated kinase 3 (PAK3), the ARF GTPase-activating proteins 1 and 2 (GIT1 and GIT2), and the Rac/Cdc42 guanine nucleotide exchange factor 6 (ARHGEF6) (Fig. 1c and Additional file 2: Figure S1A, B). Interaction of HIV-1 or SIV Nef with a similar complex comprising the paralogous PAK2 has been shown to activate the kinase activity of PAK2 and to promote Nef phosphorylation and viral replication [20, 35–38]. The connection of other common interactors (Additional file 2: Figure S1B) to HIV-1 replication and/or pathogenesis remains to be determined.

To validate selected hits from our screen, we transfected HeLa cells with plasmids encoding FTS-tagged Nef from HIV-1 or the three SIV strains, as well as the myrlysin or lyspersin subunits of BLOC-1-related complex (BORC) [39] as specificity controls. Cells were cross-linked with dithiobis succinimidyl propionate (DSP) to stabilize interactions, and solubilized FTS-tagged proteins were pulled down with Strep-Tactin beads. Endogenous proteins that co-isolated with the FTS-tagged proteins were identified by immunoblotting. We chose to perform this validation for three interactors that could be detected by commercially available antibodies and that were of particular interest to us. PAK3 was chosen because of its homology to the Nef kinase PAK2 [20, 35–38]. The serine palmitoyl transferase long chain base subunit 2 (SPTLC2) was chosen because it is involved in the biosynthesis of sphingolipids, which are important contributors to HIV-1 infectivity [40, 41]. Finally, the transmembrane autophagy-related protein 9A (ATG9A) was chosen because of the reported connections of Nef to autophagy [22–24]. Our pulldown-immunoblotting experiments revealed that PAK3, SPTLC2 and ATG9A indeed co-isolated with all four Nefs (Fig. 1e), even though in the original TAP-MS PAK3 only co-purified with SIVcpz and SIVmac, SPTLC2 with HIV-1 and SIVmac, and ATG9A with HIV-1 (Fig. 1c and Additional file 2: Figure S1). PAK3, SPTLC2 and ATG9A were pulled down to a much lesser extent with tagged myrlysin and lyspersin (Fig. 1e), supporting the specificity of the interactions with the four Nefs. The fact that these proteins were not co-isolated with all four Nefs in the TAP-MS experiment could be explained by the use of a cross-linking agent in the pulldown experiments and by the higher sensitivity of immunoblotting.

### Nef does not affect ATG9A localization, ATG9A levels and autophagy

**Fig. 2:**
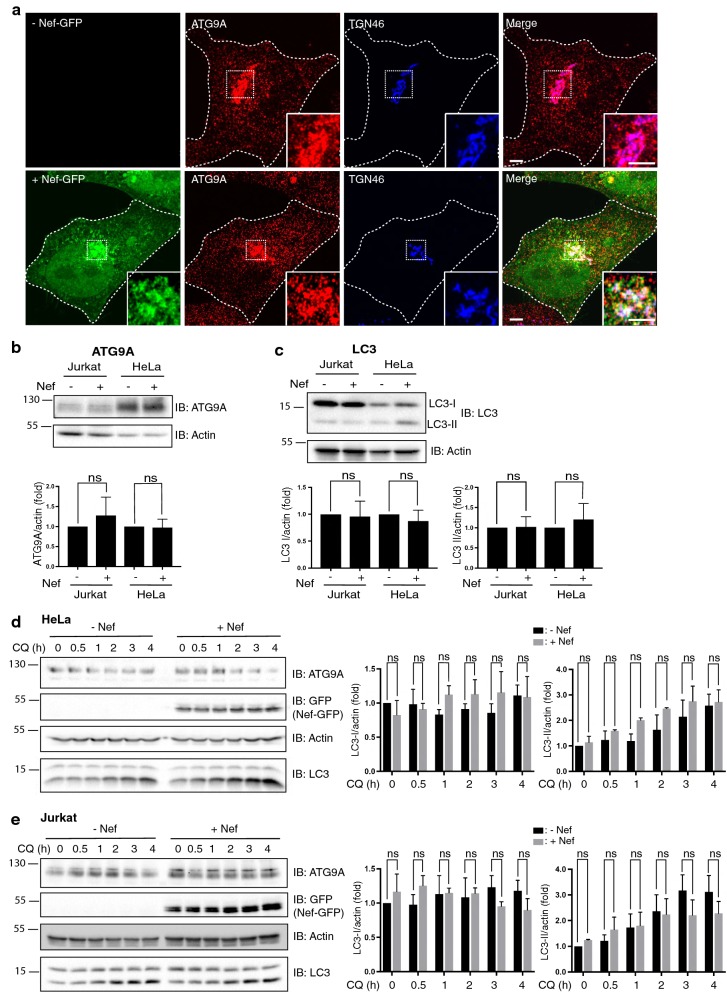
Expression of Nef does not affect ATG9A localization, ATG9A levels or autophagy. **a** HeLa cells, control (−) or transiently expressing HIV-1 Nef-GFP (+), were fixed, permeabilized, immunostained with antibodies to endogenous ATG9A or TGN46, and imaged by confocal microscopy. Scale bar: 10 μm. Insets show enlarged views of the boxed areas. **b**, **c** Lysates of Jurkat or HeLa cells stably expressing GFP (−) or HIV-1 Nef-GFP (+) were subjected to SDS-PAGE and immunoblotting with antibodies to ATG9A, LC3 or actin. The positions of molecular mass markers (in kDa) are indicated on the left. ATG9A and LC3 levels were quantified relative to actin levels. Bar graphs represent the mean ± SD from four independent experiments. Values were normalized to the ATG9A/actin ratio in the absence of Nef. Statistical significance was determined using an unpaired Student’s t-test; ns: not significant (*p* > 0.05). **d**, **e** HeLa (**d**) or Jurkat (**e)** cells, stably expressing GFP (−Nef) or Nef-GFP (+Nef), were treated for different times with 10 μM chloroquine (CQ). Cell lysates were analyzed by SDS-PAGE and immunoblotting with antibodies to the indicated proteins. The positions of molecular mass markers (in kDa) are indicated on the left. Bar graphs represent the mean ± SD of the levels of LC3-I and LC3-II relative to the level of actin from three independent experiments. Values were normalized to the levels at time 0 in the absence of Nef. Statistical significance was analyzed using a one-way ANOVA followed by a Holm-Šídák post hoc test; ns: not significant (*p* > 0.05)

Of the hits in our screen, we chose to focus on ATG9A because it had not been previously shown to interact with Nef, and because of the reported roles of autophagy in the HIV-1 replication cycle [42]. In addition, this was a protein that was already under study in our laboratory for other reasons [43, 44]. ATG9A is a multispanning membrane protein with N-terminal and C-terminal tails facing the cytosol [45]. Because Nef functions to downregulate transmembrane proteins such as CD4, MHC class I, CXCR4, CCR5 and SERINC3/5 from the surface of host cells [11–18, 46], we examined the effect of transiently expressing HIV-1 Nef-GFP on the localization and levels of endogenous ATG9A in HeLa cells. Immunofluorescence microscopy showed that, in the absence of Nef, endogenous ATG9A was distributed throughout the cytoplasm, although with a higher concentration at the *trans*-Golgi network (TGN), as detected by co-staining for TGN46 (Fig. 2a). This distribution was in line with the reported cycling of ATG9A between the TGN and peripheral autophagosomal structures [45, 47]. HIV-1 Nef-GFP exhibited a similar localization to the TGN and peripheral structures in addition to the plasma membrane (Fig. 2a), as previously reported [16, 48–50]. The co-localization of ATG9A with Nef was consistent with the interaction of these proteins detected by TAP-MS and Strep-Tactin pulldown (Fig. 1). However, immunofluorescence microscopy showed that expression of Nef-GFP did not alter the overall localization and staining intensity of ATG9A (Fig. 2a). Moreover, immunoblot analysis revealed that stable expression of HIV-1 Nef-GFP in both HeLa and Jurkat cells had no effect on the levels of endogenous ATG9A (Fig. 2b). Thus, unlike the downregulation of surface CD4, MHC class I, CXCR4, CCR5 and SERINC3/5, the intracellular localization and levels of ATG9A were unaltered by expression of Nef.

ATG9A participates in the early stages of autophagy, presumably by delivering lipids to phagophore assembly sites (PAS) [51, 52]. This function of ATG9A promotes the conversion of the autophagy protein LC3 (orthologous to yeast Atg8) from a soluble, cytosolic form (LC3-I) to a lipidated, membrane-bound form (LC3-II) [53]. This conversion can be monitored by SDS-PAGE and serves as an indicator of autophagic activity. Using this assay, we found that stable expression of HIV-1 Nef-GFP did not significantly alter the ratio of endogenous LC3-I and LC3-II to actin in either HeLa or Jurkat cells (Fig. 2c). In line with this observation, stable expression of Nef-GFP also had no effect on the punctate appearance and staining intensity of endogenous LC3-II in the cytoplasm of either HeLa or Jurkat cells (Additional file 2: Figure S2). Similarly, transient expression of Nef-GFP did not alter the number of LC3 puncta per cell (unpublished observations). After fusion of autophagosomes with lysosomes to form autolysosomes, LC3-II is degraded by lysosomal acid hydrolases [54]. Incubation of cells with chloroquine inhibits the hydrolases, resulting in accumulation of LC3-II in autolysosomes and thus providing a measure of “autophagic flux” [55]. Indeed, we found that incubation of both HeLa (Fig. 2d) and Jurkat cells (Fig. 2e) with chloroquine resulted in a time-dependent increase in the levels of LC3-II, without alteration in the levels of LC3-I. Stable expression of HIV-1 Nef-GFP in these cells had little or no effect on the kinetics and extent of LC3-II increase, or on the steady levels of LC3-I, upon chloroquine treatment (Fig. 2d, e). Therefore, expression of Nef does not affect autophagic flux. Further analyses showed that stable expression of Nef-GFP slightly increased the levels of beclin-1 but did not alter the levels of other components of the autophagy machinery, including ATG7, ATG5, ATG12, ATG16, LAMP-1 and SNAP29 in either HeLa or Jurkat cells (Additional file 2: Figure S3). The fact that there were no differences in autophagic flux, however, indicated that the increase in beclin-1 levels induced by the co-expression of HIV-1 Nef had no impact on the process. From these experiments, we concluded that Nef does not cause major alterations in autophagy in HeLa and Jurkat cells despite its interaction with ATG9A.

### The intracellular distribution of Nef is independent of ATG9A and autophagy induction

We next addressed the converse possibility that ATG9A influenced the intracellular localization of Nef. To this end, we used the CRISPR/Cas9 system to inactivate the ATG9A gene in both HeLa and Jurkat cells. Immunoblot analysis confirmed the absence of ATG9A protein in the KO cells (Additional file 2: Figure S4A, C). HIV-1 Nef-GFP was then stably expressed in these cells and its intracellular localization determined by confocal microscopy. We observed that ATG9A KO did not affect the levels (Additional file 2: Figure S4A, C) and intracellular distribution (Additional file 2: Figure S4B, D) of Nef-GFP in either HeLa or Jurkat cells. In addition, we tested if the distribution of stably expressed Nef-GFP in HeLa and Jurkat cells was affected by induction of autophagy by treatment with the mTORC1 inhibitor Torin1 or by incubation in amino-acid- and serum-free medium. We observed that, while these treatments increased the number of cytoplasmic LC3-II puncta (Additional file 2: Figure S5B, D), they had no effect on the overall distribution of GFP-Nef (Additional file 2: Figure S5A, C). From these experiments we concluded that the intracellular distribution of Nef-GFP is independent of ATG9A and of autophagy induction.

### ATG9A promotes HIV-1 infectivity

**Fig. 3:**
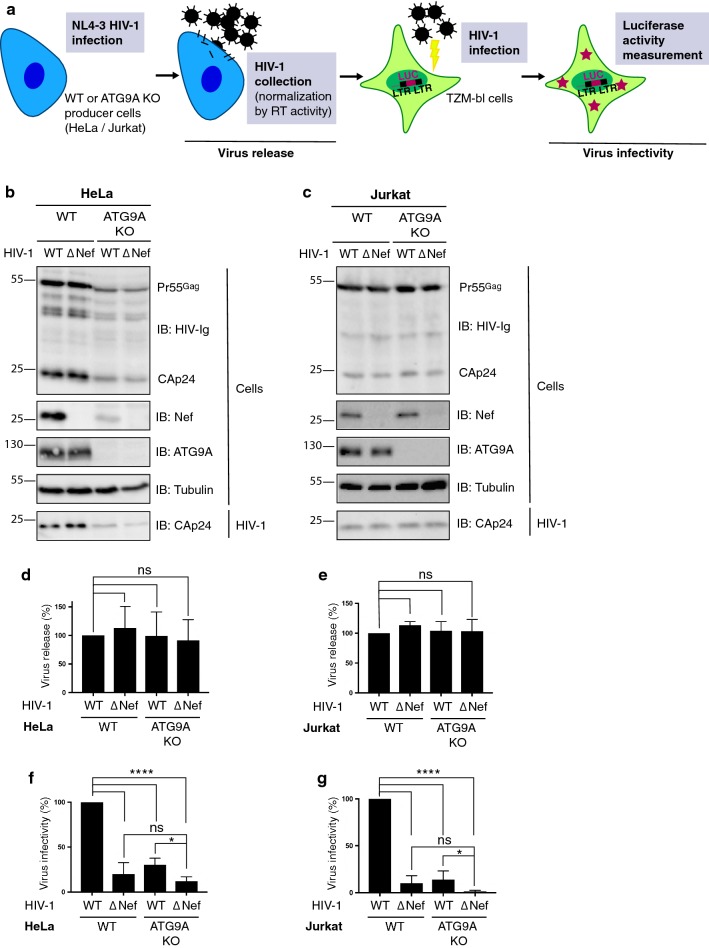
ATG9A is required for HIV-1 infectivity. **a** Flowchart representing the steps to measure HIV-1 release and infectivity. Briefly, RT-normalized HIV-1 NL4-3 particles, produced from WT or ATG9A-KO HeLa or Jurkat cells, were used to infect TZM-bl cells. These cells express the LUC (luciferase) reporter gene under control of the HIV-1 long terminal repeat (LTR). Reporter gene expression is proportional to the number of infectious particles present in the initial inoculum. **b**, **c** WT and ATG9A-KO HeLa (**b**) and Jurkat (**c**) cells were infected with WT or Nef-defective (ΔNef) pNL4-3 for 8 h and the medium was replaced. Two days post-infection, the culture supernatant was filtered, and viruses were collected by ultracentrifugation. Cell and virus lysates were analyzed by SDS-PAGE and immunoblotting with anti-HIV immunoglobulin (HIV-Ig) to detect viral proteins (Pr55^Gag^, CAp24), and with antibodies to HIV-1 Nef, ATG9A and tubulin. The positions of molecular mass markers (in kDa) are indicated on the left. **d**, **e** Virus release was quantified by determining the amount of virion-associated CAp24 relative to the level of total CAp24 in cells and viruses. Bar graphs represent the mean ± SD from four (**d**) or five (**e**) independent experiments. Values were normalized to the release of WT HIV-1 from WT cells. Statistical significance was calculated using a one-way ANOVA test; ns: not significant (*p* > 0.05). **f**, **g** RT-normalized HIV-1 virus stocks were used to infect TZM-bl cells. Infection of reporter cells was measured by detection of LUC activity at 2 days post-infection. Values were normalized to the infectivity of WT HIV-1 from parental cells. Data were evaluated for statistical significance by using a one-way ANOVA test (ns: *p* > 0.05, *p < 0.05, ****p < 0.0001)

Because Nef and ATG9A did not affect each other’s levels or intracellular distribution, and Nef did not detectably alter autophagy, we focused our subsequent studies on the possibility that ATG9A could mediate the effects of Nef on the HIV-1 replication cycle independently of its role in autophagy. To address this question, we examined the release and infectivity of WT and Nef-deleted (ΔNef) HIV-1 particles produced in WT and ATG9A-KO HeLa and Jurkat cells (Fig. 3a). The procedure consisted of infecting HeLa or Jurkat cells with reverse-transcriptase (RT)-normalized amounts of WT or ΔNef HIV-1 particles pseudotyped with vesicular stomatitis virus G glycoprotein (VSV-G). After 8 h of infection, cells were washed and cultured in fresh medium. Two days later, the culture supernatant was filtered, and a portion was centrifuged to collect viruses. Viral and cell lysates were immunoblotted with antibodies to viral proteins to quantify the efficiency of virus release (Fig. 3b, c). We noticed that the production of HIV-1 proteins was reduced in ATG9A-KO relative to parental HeLa cells for both WT and Nef-defective virus (Fig. 3b); this was not the case in Jurkat cells (Fig. 3c). While we do not know the reason for this difference, the key observation in these experiments was that the ratio of released and cell-associated capsid protein CAp24 was unchanged by either ATG9A KO or Nef deletion (Fig. 3d, e). Therefore, we concluded that these mutations do not affect the efficiency of virus release. We subsequently used equal numbers of virus particles, normalized by reverse transcriptase (RT) activity, to infect CD4-positive TZM-bl cells, which express the luciferase (LUC) reporter gene under control of the HIV-1 long terminal repeat (LTR). Measurement of LUC activity in these cells allowed calculation of viral infectivity. Using this procedure, we observed that deletion of Nef decreased the infectivity of the virus released from WT HeLa and Jurkat cells to ~ 20% and ~ 10%, respectively, of the WT virus control (Fig. 3f, g), as previously reported [56–60]. The greater effect of Nef deletion on HIV-1 infectivity in Jurkat cells relative to HeLa cells was in line with previous findings, and was likely due to the higher levels of SERINC3/5 expression in Jurkat cells [14]. Importantly, KO of ATG9A also reduced infectivity of the WT virus to ~ 30% and ~ 14% of the control in HeLa and Jurkat cells respectively (Fig. 3f, g). Combining Nef deletion with ATG9A KO resulted in a further reduction of infectivity to ~ 12% and ~ 1% of the control from HeLa and Jurkat cells and WT virus, respectively (Fig. 3f, g). The difference between the infectivity of WT and ΔNef virus in ATG9A-KO cells was statistically significant (p < 0.05), indicating that the effects of Nef deletion and ATG9A KO are additive, and that ATG9A contributes to the infectivity of both WT and Nef-deleted HIV-1.

### Nef-ATG9A interaction is not required for HIV-1 infectivity

**Fig. 4:**
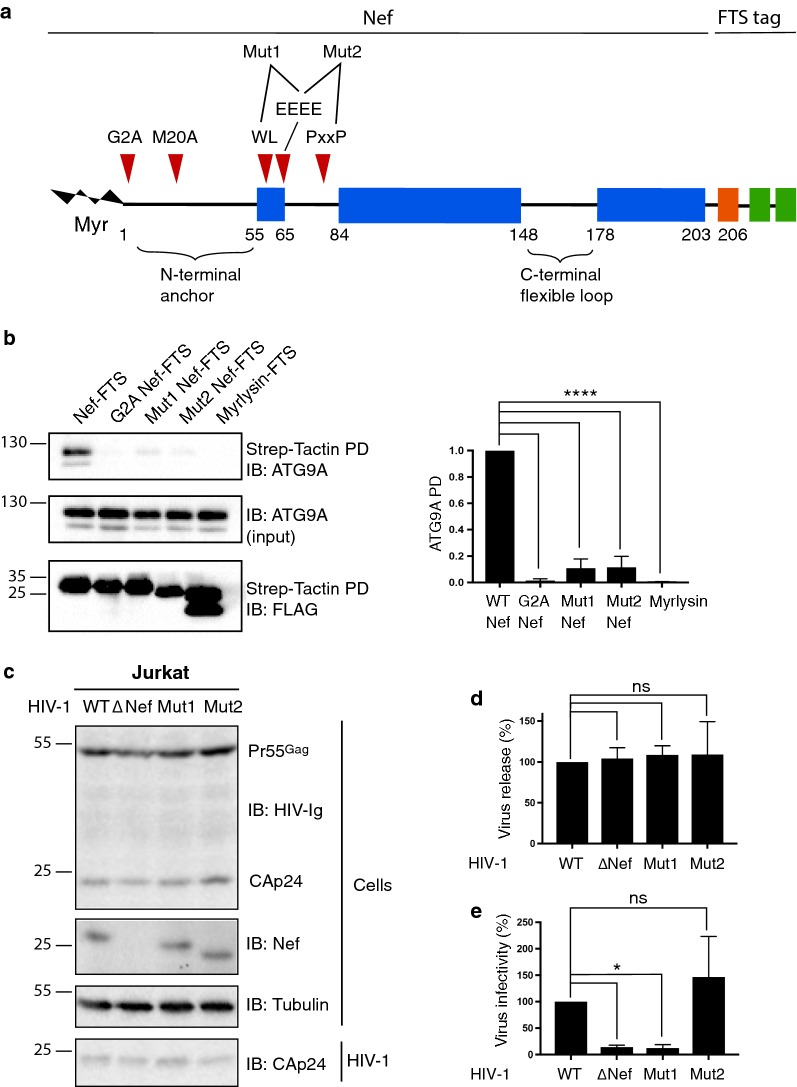
ATG9A-Nef interaction is not required for HIV-1 infectivity. **a** Schematic representation of HIV-1 NL4-3 Nef as described in Fig. 1a. Red arrowheads indicate residues that were mutated to alanine. Mut1 represents mutation of WL-EEEE to AA-AAAA and Mut2 mutation of EEEE-PxxP to AAAA-AxxA. **b** HeLa cells were transfected with plasmids encoding WT HIV-1 Nef-FTS, Nef-FTS mutants (G2A, Mut1, or Mut2) or Myrlysin-FTS, and cell extracts were incubated with Strep-Tactin beads. The isolated proteins were subjected to SDS-PAGE and immunoblotting with antibodies to FLAG and ATG9A. The positions of molecular mass markers (in kDa) are indicated on the left. The amount of ATG9A in the isolated samples was quantified relative to the amount of ATG9A in the input. Values were normalized to the ATG9A PD/input ratio in the WT condition. Bar graphs represent the mean ± SD from three independent experiments. Statistical significance was analyzed using a one-way ANOVA test (****p < 0.0001). **c** RT normalized WT and Nef-mutant NL4-3 viruses produced in HEK-293T cells were used to infect Jurkat cells for 8 h, and the medium was replaced. At 48 h post-infection, cell and virus fractions were harvested and analyzed by immunoblotting with anti-HIV immunoglobulin (HIV-Ig), and antibodies to Nef and tubulin. In **b** and **c**, the positions of molecular mass markers (in kDa) are indicated on the left. **d** Virus release was quantified by determining the amount of virion-associated CAp24 relative to the total amount of CAp24 in cells and virions. Bar graphs represent the mean ± SD from four independent experiments. Statistical significance was analyzed using a one-way ANOVA test; ns: not significant (*p* > 0.05). **e** RT-normalized HIV-1 particles were used to infect TZM-bl cells. Infection was determined by detection of LUC activity 2 days post-infection. Data were evaluated for statistical significance using a one-way ANOVA; ns: not significant (*p* > 0.05), *p < 0.01

We next addressed the question of whether the physical interaction of HIV-1 Nef with ATG9A is important for HIV-1 infectivity. To this end, we first sought to identify mutations in Nef that prevented its interaction with ATG9A. Among the mutations tested were glycine-2 to alanine (G2A) [61–64], methionine-20 to alanine (M2A) [65], tryptophan-58 and leucine-59 (WL) to two alanines (AA) [66], glutamate-65, -66, -67 and -68 (EEEE) to four alanines (AAAA) [67, 68] and proline-72 and -75 (PxxP) to two alanines (AxxA) [21] (Fig. 4a). The G2A mutation prevents myristoylation of Nef, abrogating its association with membranes and most of its biological functions [61–64]. The other mutations were previously reported to prevent association of Nef with various cellular proteins [65–69]. Pulldown assays such as those shown in Fig. 1e demonstrated that, while all mutations partially reduced interaction of transiently transfected Nef-FTS with endogenous ATG9A in HeLa cells, only the G2A mutation completely abrogated the interaction (Fig. 4b and Additional file 2: Figure S6). This finding is consistent with the Nef-ATG9A interaction occurring in association with membranes. We also tested the effects of combining two of the above mutations and found that the WL-EEEE (i.e., Mut1) and EEEE-PxxP (i.e., Mut2) mutations further reduced the Nef-ATG9A pulldown to ~ 10% that of WT Nef (Fig. 4b). These findings indicated that the region between Nef residues 58 and 75 is particularly important for interaction with ATG9A.

To test whether inhibition of Nef-ATG9A binding prevented the enhancement of HIV-1 infectivity by Nef, we performed HIV-1 release and infectivity assays such as those shown in Fig. 3 using Nef mutants. We found that WT, ΔNef, Mut1 and Mut2 viruses were released with similar efficiency from Jurkat cells (Fig. 4c, d). Interestingly, Mut1 exhibited decreased infectivity to the same extent as ΔNef (Fig. 4e), indicating that the WL and EEEE motifs are important for this function of Nef, as they are for CD4 [66] and MHC-I downregulation [68], respectively. However, Mut2 was as infectious as the WT virus (Fig. 4e), breaking the correlation between the Nef-ATG9A interaction and infectivity. From these results, we concluded that ATG9A is required for optimal infectivity of HIV-1 independently of its interaction with Nef.

### ATG9A is dispensable for SERINC5 downregulation by Nef

**Fig. 5:**
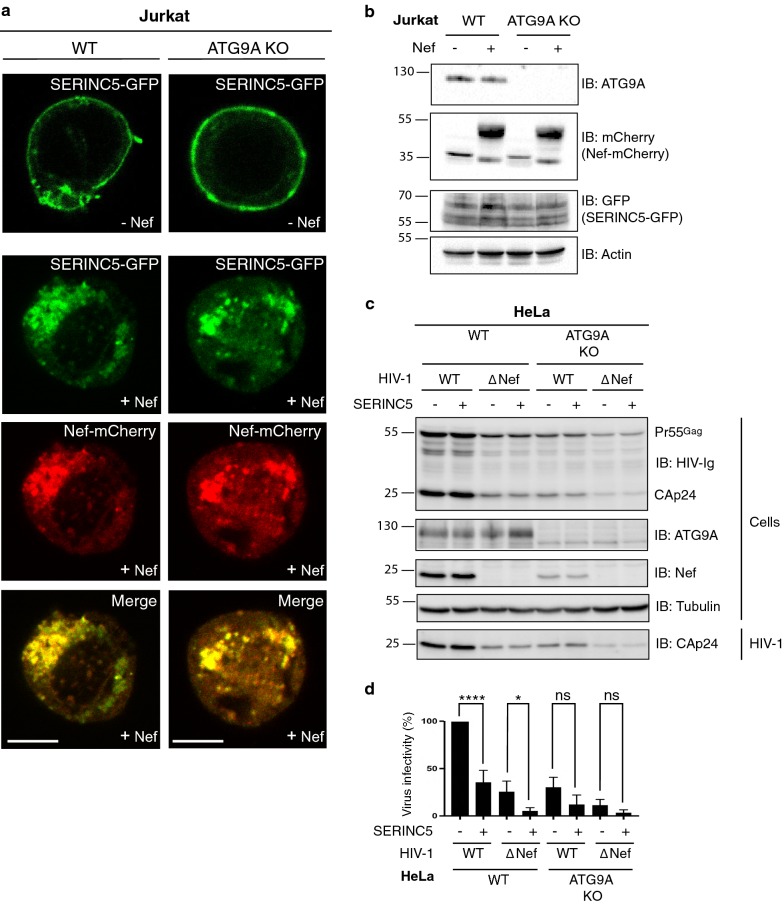
ATG9A is dispensable for SERINC5 downregulation by Nef. **a** WT and ATG9A-KO Jurkat cells transiently expressing SERINC5-GFP minus (−) or plus (+) Nef-mCherry were fixed, permeabilized, and imaged by confocal microscopy. Scale bars: 10 μm. **b** Lysates of WT or ATG9A-KO Jurkat cells, transiently expressing mCherry (−) or HIV-1 Nef-mCherry (+) in addition to SERINC5-GFP, were subjected to SDS-PAGE and immunoblotting with antibodies to ATG9A, mCherry, GFP or actin. The positions of molecular mass markers (in kDa) are indicated on the left. The different bands detected by the GFP antibody correspond to different glycosylation forms of SERINC5 [17]. **c** WT and ATG9A-KO HeLa cells were co-transfected with 50 ng of SERINC5 and 1 μg of WT or ΔNef pNL4-3 for 6–8 h and the medium was replaced. One day later, the culture supernatant was filtered, and virus pellets were collected by ultracentrifugation. Cell and virus lysates were analyzed by SDS-PAGE and immunoblotting with HIV-Ig to detect viral proteins (Pr55^Gag^, CAp24), and with antibodies to HIV-1 Nef, ATG9A and tubulin. In **b** and **c**, the positions of molecular mass markers (in kDa) are indicated on the left. **d** Infectivity of virions was measured in TZM-bl cells as in Fig. 3. Values were normalized to the infectivity of WT HIV-1 from parental HeLa cells in the absence of SERINC5. Data were evaluated for statistical significance by using a one-way ANOVA followed by a Holm-Šídák post hoc test; ns: not significant (*p* > 0.05), *p < 0.05, ****p < 0.0001)

We hypothesized that the Nef-independent function of ATG9A might involve downregulation of a host-cell restriction factor that impairs HIV-1 infectivity. In this regard, Nef was shown to increase HIV-1 infectivity by antagonizing the restriction factors SERINC3 and SERINC5 in the host cells [14–18, 65]. This antagonism was shown to involve downregulation of SERINC3/5 from the cell surface, preventing its incorporation into budding virions and thus its inhibitory effect on fusion of virions with target cells [14–18]. To test our hypothesis, we examined the Nef-induced downregulation of SERINC5-GFP in WT and ATG9A-KO Jurkat cells. Confocal microscopy analyses showed that in WT cells HIV-1 Nef-mCherry induced redistribution of SERINC5-GFP from the plasma membrane to an intracellular compartment where the two proteins co-localized (Fig. 5a, b). KO of ATG9A had no effect on this redistribution, as well as on the expression level of SERINC5 (Fig. 5a, b). These experiments demonstrated that SERINC5 downregulation by Nef is independent of ATG9A. Further experiments showed that overexpression of SERINC5 had no effect on virus release (Fig. 5c), but reduced the infectivity of WT and ΔNef viruses in WT cells (Fig. 5d). SERINC5 overexpression also reduced the infectivity of WT and ΔNef viruses in ATG9A-KO cells, although the differences were not statistically significant (Fig. 5d).

### The requirement of ATG9A for HIV-1 infectivity is dependent on the Env glycoprotein

**Fig. 6:**
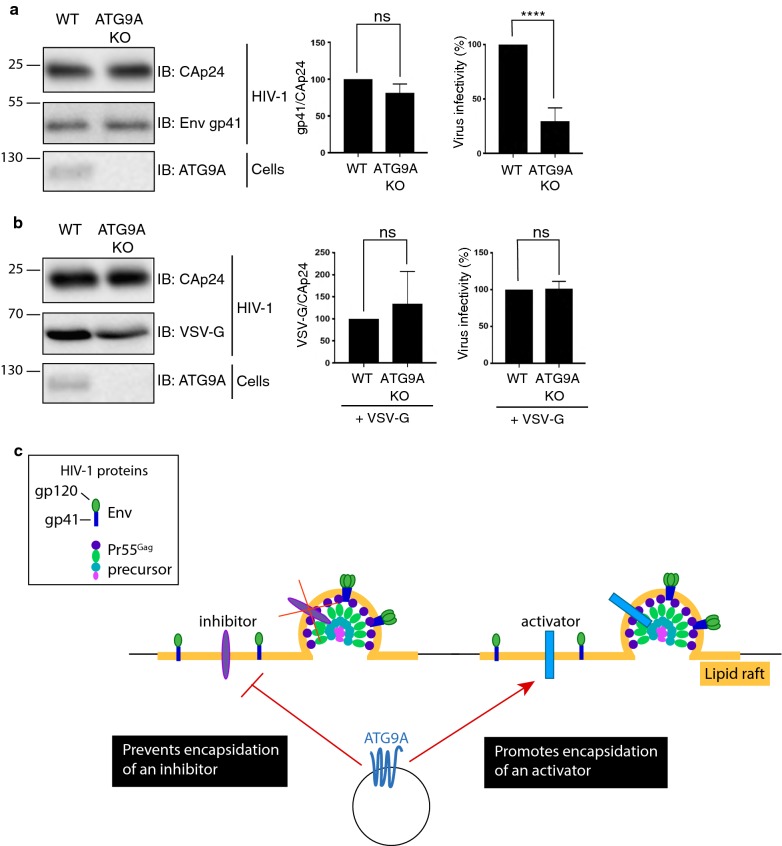
Env-dependence of ATG9A role in HIV-1 infectivity. **a** WT HIV-1 particles, produced from WT and ATG9A-KO HeLa cells, and cell lysates were analyzed by SDS-PAGE and immunoblotting with HIV-Ig to detect CAp24, and with antibodies to gp41 and ATG9A. The positions of molecular mass markers (in kDa) are indicated on the left. The incorporation of gp41 was quantified by determining the amount of virion-associated gp41 relative to CAp24. RT-normalized HIV-1 virus stocks were used to infect TZM-bl cells. Infection of reporter cells was measured by detection of LUC activity 2 days post-infection. Values were normalized to parental HeLa cells. Statistical significance from three independent experiments was determined using an unpaired Student’s t-test; ns: not significant (*p* > 0.05), ****p < 0.0001). **b** VSV-G pseudotyped HIV-1 particles were produced from WT and ATG9A-KO HeLa cells, and cell lysates were analyzed by SDS-PAGE and immunoblotting with HIV-Ig to detect CAp24, and with antibodies to VSV-G and ATG9A. The positions of molecular mass markers (in kDa) are indicated on the left. The incorporation of VSV-G was quantified by determining the amount of virion-associated VSV-G relative to CAp24. RT-normalized HIV-1 particles were used to infect TZM-bl cells. Virus infectivity was determined as in panel B. **c** Model for the role of ATG9A in HIV-1 infectivity. ATG9A promotes HIV-1 infectivity independently of Nef. ATG9A could promote the removal of an inhibitor or the incorporation of an activator of the infectivity of the viral particles. This effect of ATG9A on HIV-1 infectivity is dependent on HIV-1 Env, as pseudotyping with VSV-G overcomes the infectivity defect

HIV-1 infection is dependent on the binding of Env to CD4 and coreceptors on target cells. HIV-1 Env is synthesized as a gp160 precursor that is proteolytically processed to generate the surface gp120 and transmembrane gp41 subunits [70]. Incorporation of Env into virions is essential for HIV-1 infectivity [71]. Because ATG9A KO impairs HIV-1 infectivity, we evaluated whether ATG9A is required for Env incorporation into virions by performing immunoblotting for gp41 in virus particles released from WT and ATG9A-KO HeLa cells. We observed that, despite the requirement of ATG9A for optimal infectivity, ATG9A KO did not alter the levels of gp41 relative to CAp24 present in the released virions (Fig. 6a). To investigate whether the requirement of ATG9A for infectivity was nevertheless dependent on Env, we examined the infectivity of HIV-1 pseudotyped with the VSV-G glycoprotein produced in WT and ATG9A-KO cells. These experiments showed equal levels of VSV-G and CAp24 in viruses released by WT and ATG9A-KO cells (Fig. 6b), demonstrating that ATG9A does not affect incorporation of VSV-G glycoprotein into the virus. Importantly, the VSV-G-pseudotyped viruses produced in WT and ATG9A-KO cells were equally infections in the indicator TZM-bl cells (Fig. 6b). Thus, replacement of HIV-1 Env with VSV-G overcame the requirement of ATG9A for optimal infectivity. This finding indicates that the ATG9A requirement in HIV-1 infectivity is Env-dependent.

## Discussion

The main conclusion of our study is that the host cell protein ATG9A is required for HIV-1 infectivity in an Env-dependent manner. We arrived at this conclusion by a rather circuitous route that started with TAP-MS experiments aimed at identifying cellular interactors for the Nef proteins of HIV-1 and SIV. This approach resulted in the identification of dozens of interactors, some known and some novel. Among the novel interactors was ATG9A, which had not been previously implicated in HIV-1 replication or pathogenesis. The interaction of HIV-1 Nef with ATG9A was specific in that ATG9A was never identified in 411 other TAP-MS experiments recorded in the CRAPome database, and control proteins such as myrlysin and lyspersin did not significantly pull down ATG9A (Figs. 1e, 4b). Furthermore, G2A, WL-EEEE and EEEE-PxxP mutations in Nef abrogated the pulldown of ATG9A (Fig. 4b). These findings prompted us to analyze the biological significance of the Nef-ATG9A interaction.

Because ATG9A is an essential component of the core autophagy machinery, we investigated whether Nef could alter autophagy via interaction with ATG9A. Previous studies have reported multiple connections between HIV-1 infection and autophagy [22, 72–75]. In particular, Nef has been shown to perturb autophagy in both infected and bystander cell types, including macrophages, cardiomyocytes, astrocytes, mesenchymal cells and HeLa cells [22, 24, 76–81]. In our experiments, using HeLa cells and Jurkat T cells, however, Nef did not alter the intracellular localization or levels of ATG9A (Fig. 2a, b). Moreover, Nef had no effect on the number of LC3 puncta and the levels of LC3-I and LC3-II in control or chloroquine-treated cells (Fig. 2c–e), indicating that neither autophagy initiation nor autophagic flux were affected by HIV-1 Nef. It is noteworthy that only one of the previous studies used HeLa cells, reporting a doubling of the number of LC3 puncta per cell upon Nef expression [76]. We do not know the reason for the difference with our results, although we should point out that our analyses were more extensive, including biochemical measurement of the levels of LC3 species. None of the previous studies used Jurkat T cells, which are a particularly relevant cell type for HIV-1 infection. From these observations, we concluded that, in the cells and conditions used in our studies, Nef had no discernible effect on ATG9A and autophagy.

Because Nef promotes HIV-1 infectivity through downregulation of restriction factors such as SERINC3/5 [14–18], we next examined the possibility that ATG9A could play a role in infectivity. Indeed, we found that ablation of the *ATG9A* gene in host cells reduced infectivity to a similar extent as deletion of the Nef gene in the virus (Fig. 3). However, additional observations indicated that these effects of Nef and ATG9A were independent. First, the decreases in HIV-1 infectivity caused by Nef deletion and ATG9A KO were additive (Fig. 3d). In other words, deletion of Nef further attenuated the already reduced infectivity of HIV-1 produced in ATG9A-KO cells. Moreover, mutation of EEEE-PxxP residues in Nef abrogated interaction with ATG9A but did not alter infectivity (Fig. 4). Hence, the interaction of Nef with ATG9A identified in our initial experiments is not required for Nef to enhance HIV-1 infectivity. In line with this conclusion, ATG9A KO did not prevent Nef from downregulating SERINC5 from the cell surface (Fig. 5), a well-established function of Nef in enhancement of HIV-1 infectivity.

Our experiments nonetheless led to the discovery that, independently of its interaction with Nef, ATG9A itself promotes infectivity. We speculate that ATG9A could do so by participating in the removal of a factor that inhibits infectivity, as previously demonstrated for the Nef-induced downregulation of SERINC3/5 from the surface of the host cells [14–18]. This factor, however, is unlikely to be SERINC5 because the absence of ATG9A did not change the intracellular distribution of SERINC5-GFP or prevent the Nef-induced redistribution of plasma membrane SERINC5-GFP to intracellular compartments (Fig. 5). Because the requirement of ATG9A is independent of Nef, we cannot rule out the alternative possibility that ATG9A promotes the incorporation of a positive factor into the budding virions. In any event, these hypothetical regulators would act through Env, as the requirement of ATG9A for particle infectivity can be overridden by substitution of HIV-1 Env with VSV-G (Fig. 6b). Because ATG9A is thought to be a lipid transporter [51, 52], it could perhaps contribute to creating a lipid environment in the viral envelope that promotes Env- but not VSV-G-mediated fusion with the host cell membrane. Testing this hypothesis will require the determination of the protein and lipid composition of HIV-1 particles produced in the presence or absence of ATG9A.

## Conclusions

In summary, the present study (1) identified novel interactors of several Nefs, including ATG9A, and (2) demonstrated a requirement of ATG9A in the HIV-1-producing cells for optimal infectivity of the viral progeny. Although one set of findings led to the other, the interaction of ATG9A with Nef and its role in the promotion of viral infectivity turned out to be unrelated phenomena, i.e., Nef and ATG9A independently contribute to enhanced HIV-1 infectivity. Nevertheless, these findings should enable further studies of Nef and ATG9A function in HIV-1 propagation. The novel Nef interactors identified here could mediate some of the known effects of Nef on HIV-1 replication, infectivity and pathogenesis. The other two hits that were further validated in pulldown assays, PAK3 and SPTLC2, are particularly good candidates for mediating Nef actions. The requirement of ATG9A additionally points to a role of this protein in the production of infectious HIV-1 particles, possibly by a mechanism that is distinct from its role in autophagy.

## Materials and methods

### Plasmids and mutagenesis

The Nef proteins from the HIV-1 NL4-3 strain and from three SIV strains (cpz GAB1, mac239 and smm FWR1), tagged with a C-terminal FTS tag, were cloned into a pcDNA5/FRT/TO Dox-inducible plasmid. The human ATG9A, myrlysin (also known as LOH12CR1 and BORCS5), and lyspersin (also known as C17orf59 and BORCS6), and the HIV-1 pNL4-3 Nef cDNAs were cloned into a pcDNA3.1 plasmid encoding a single C-terminal FTS tag. The cDNAs encoding human ATG9A and SERINC5, and Nef from pNL4-3, were cloned into pEGFP-N1 (Clontech). Nef from pNL4-3, was cloned into pmCherry-N1 (Clontech). The full-length infectious HIV-1 molecular clone pNL4-3 [82], and its Nef-defective (pNL4-3 ΔNef) [83] and Env-defective (pNL4-3/KFS) [84] derivatives have been described previously. The plasmid pHCMV-G [85], encoding the G glycoprotein of vesicular stomatitis virus (VSV) was kindly provided by Dr. Jane Burns (University of California, San Diego, La Jolla, CA). All mutations were generated by site directed mutagenesis (QuikChange, Agilent, or Q5^®^ Hot Start High-Fidelity 2X, NEB) and confirmed by DNA sequencing.

### Cell culture, transfection and infection

HEK-293T cells, and WT and ATG9A-KO HeLa cells, were cultured in Dulbecco-modified Eagle’s medium (DMEM) containing 10% fetal bovine serum (FBS) and 2 mM l-glutamine at 37 °C, 5% CO_2_. Transient plasmid transfection of HeLa cells was performed using Lipofectamine 2000 (Invitrogen) according to the manufacturer’s instructions. Immunoblot analysis, co-immunoprecipitations and immunofluorescence microscopy experiments were done 24 h after transfection. WT and ATG9A-KO Jurkat E6.01 cells were grown in Roswell Park Memorial Institute 1640 (RPMI-1640) medium containing 10% FBS and 2 mM l-glutamine at 37 °C, 5% CO_2_. Jurkat cells were infected for 72 h with lentiviral particles. Briefly, 1 million cells were centrifuged for 30 min at 1500 rpm with supernatant containing viruses in 500 μl complete medium supplemented with 2.5 μg polybrene. After 4 h of incubation at 37 °C, medium was replaced with complete DMEM medium.

### Tandem affinity purification and mass spectrometry (TAP-MS)

HEK-293T cells were stably transfected with plasmids encoding the FTS-tagged HIV-1 and SIV Nef constructs and the empty FTS vector described above. Cells were lysed in 50 mM Tris-HCl (pH 7.4), 300 mM NaCl, 5 mM EDTA, and 0.5% NP-40 supplemented with proteinase inhibitor cocktail (Roche) for 30 min. Cell lysates were cleared by centrifugation at 17,000×*g* for 15 min and incubated overnight with Strep-Tactin resin (IBA) at 4 °C. Bound proteins were washed 3 times with 50 mM Tris-HCl (pH 7.4), 300 mM NaCl, 5 mM EDTA, and 0.5% NP-40 and eluted with 2.5 mM desthiobiotin. Proteins were further purified using FLAG M2 antibody-coated beads (Sigma). After incubation for 3 h at 4 °C, samples were washed 3 times as previously described and eluted twice with 500 μl of 500 μg/ml 3 × FLAG peptide (Sigma). Proteins were precipitated with 10% trichloroacetic acid (TCA) for 15 min at − 20 °C, centrifuged for 30 min at 4 °C, washed twice with acetone and air-dried. Samples were analyzed by liquid chromatography (LC)/MS at the Taplin MS facility (Harvard Medical School).

### Filtering and analysis of mass spectrometry data

Initial datasets were filtered against the experimental control of the vector alone, and proteins with two or more unique peptides (i.e., number of peptides that can be assigned as being unique to that protein) were selected for analysis. To remove additional contaminants not present in the negative control, the datasets underwent a second filtering using the Contaminant Repository for Affinity Purification Mass Spectrometry Data (CRAPome, www.crapome.org). Two values from the CRAPome database were used from the database to filter the dataset: the “average spectral count” (AveSC), which is the average number of peptides in the CRAPome control experiments where the protein appeared, and the “experiment appeared” (ExpAP) ratio, which is the number of times that protein appeared in a CRAPome control experiment divided by the total number of CRAPome experiments analyzed. By multiplying AveSC and ExpAP, we obtained an estimate of both the number of times the protein was identified in a CRAPome negative control and its abundance when it was identified in one of the CRAPome control experiments. If this value was lower than the threshold (set at 0.025), the proteins were assigned as specific hits in our analysis, as we considered them as not frequently or abundantly appearing in the CRAPome database of negative control experiments. The BioGRID database was used to map protein–protein interactions. Positive interactions were mapped as connected nodes. The CRAPome filtering and BioGRID analyses were performed using the respective APIs in Python 3.7.2 with the networks generated using NetworkX and formatted for publication using Adobe Illustrator. RPL17 was manually removed from the dataset as it was annotated as RPL17-C18orf32 in the original dataset and RPL17 in the CRAPome, leading to it being incorrectly included despite having a CRAPome score above the threshold.

### DSP cross-linking

Prior to Strep-Tactin pulldown, HeLa cells were cross-linked to stabilize protein–protein interactions. One 10-cm culture dish of cells per sample was transfected with Lipofectamine 2000 for 24 h. Cells at 90% confluency were washed twice with ice-cold PBS containing 0.1 mM CaCl_2_ and 1.0 mM MgCl_2_ (PBSCM) buffer. DSP (ThermoFisher) was dissolved in DMSO before dilution into PBSCM buffer and addition to the cell suspension at 1 mM final concentration. Cross-linking was performed at 4 °C for 6 h. The DSP reaction was stopped by removing the DSP solution and adding 1 × ice-cold DSP quenching solution (20 mM Tris pH 7.4 in PBSCM buffer) at 4 °C for 15 min. Cells were washed twice with ice-cold PBSCM buffer prior to lysis.

### Strep-Tactin precipitation

Cells were lysed in 50 mM Tris-HCl (pH 7.4), 300 mM NaCl, 5 mM EDTA, and 0.5% NP-40 supplemented with proteinase inhibitor cocktail (Roche) for 30 min. Cell lysates were cleared by centrifugation at 17,000×*g* for 15 min and incubated overnight with Strep-Tactin resin (IBA) at 4 °C. Bound proteins were washed three times with 50 mM Tris-HCl (pH 7.4), 300 mM NaCl, 5 mM EDTA and 0.5% NP-40. Beads were resuspended with 2× NuPage LDS sample buffer (ThermoFischer) supplemented with 50 mM DTT and incubated at 37 °C for 15 min. Beads were pelleted by centrifugation and eluates subsequently analyzed by SDS-PAGE.

### Antibodies

We used the antibodies in parentheses to the following antigens: ATG9A (Abcam, catalog #108338), FLAG M2 (Sigma, F1804), PAK3 (Abnova, PAB2300), SPTLC2 (Abcam, ab23696), GFP-HRP (MACS, 130091833), actin-HRP (Sigma, A3854), beclin 1 (Cell Signaling, 3738), ATG7 (Cell Signaling, 8558), ATG5 (Cell Signaling, 12994), ATG12 (Cell Signaling, 4180), ATG16 (Cell Signaling, 8089), LC3 (Cell Signaling, 3868), LAMP-1 (Cell Signaling, 9091), SNAP29 (Abcam, 138500), gp41 (NIH AIDS Reagent Program, 2F5), TGN46 (Bio-Rad, AHP500G), anti-HIV immunoglobulin (NIH AIDS Reagent Program, HIV-Ig), Nef (NIH AIDS Reagent Program, 2949), α-tubulin (Sigma, T5168), VSV-G (Sigma, V5507), Alexa Fluor 488-conjugated donkey anti-rabbit IgG (Invitrogen, A21206), Alexa Fluor 488- conjugated donkey anti-mouse IgG (Invitrogen, A21202), Alexa Fluor 555-conjugated donkey antirabbit IgG (Invitrogen, A31572), Alexa Fluor 555-conjugated donkey anti-mouse IgG (Invitrogen, A31570), Alexa Fluor 555-conjugated donkey anti-sheep IgG (Invitrogen, A21436), HRP-conjugated donkey anti-rabbit IgG (GE Healthcare, NA934V), and HRP-conjugated sheep anti-mouse IgG (GE Healthcare, NXA931).

### SDS-PAGE and immunoblotting

Samples were incubated at 37 °C in sample buffer for 5 min (ATG9A detection) or 95 °C for 2 min and loaded onto a 10% polyacrylamide gel using 1× Tris-glycine-SDS (IPM scientific) running buffer and transferred onto a nitrocellulose membrane (Bio-Rad). Membranes were saturated with 3% milk (Bio-Rad) for 2 h at 4 °C and incubated overnight at 4 °C with the indicated primary antibodies. HRP-conjugated secondary antibodies were incubated with the membrane at room temperature for 1 h. HRP signal was detected using the Clarity or Femto ECL kit (ThermoFisher). Images were captured with the Chemidoc system (Bio-Rad). Protein bands were quantified using Imagelab-Chemidoc software (Bio-Rad).

### Immunofluorescence microscopy

Cells were cultured on coverslips in a 24-well plate, and 24 h after transfection fixed with 4% paraformaldehyde in PBSCM for 15 min at room temperature (RT). Cells were permeabilized with 0.2% Triton X-100 in PBSCM for 10 min at room temperature, except for LC3 staining in which cells were treated for 10 min with 100% methanol previously stored at − 20 °C. Primary antibodies and Alexa-conjugated secondary antibodies were diluted in 0.2% BSA-containing PBSCM to probe proteins of interest. Coverslips were mounted on glass slides with DAPI-Fluoromount-G (EMS). Confocal microscopy images were collected using a Zeiss LSM 710 confocal microscope with a Plan Apochromat 63 × objective. Image analysis was performed with Fiji software.

### Chloroquine treatment

To measure autophagic flux, HeLa and Jurkat cells were treated for different times with 10 μM chloroquine (CQ). A control condition without treatment was performed in parallel. Following incubation, cells were lysed for 15 min in 1 ml of ice-cold lysis buffer containing 300 mM NaCl, 50 mM Tris pH 7.4, 1 mM EDTA, 1% Triton X-100 and EDTA-free complete protease inhibitors (Roche). Cell lysates were collected, centrifuged at 17,000×*g* for 10 min at 4 °C and further analyzed by immunoblotting.

### Induction of autophagy

Starvation was performed by incubating HeLa and Jurkat cells in Hanks’ balanced salt solution (HBSS) for 1 h at 37 °C. Cells were briefly washed once with starvation medium before incubation. Torin1 treatment was performed by incubating cells for 2 h at 37 °C in DMEM or RPMI-1640 with 200 mM of the drug. Following treatment, cells were lysed and analyzed by immunofluorescence.

### CRISPR/Cas9 knock-out

The ATG9A gene was inactivated using the CRISPR/Cas9 system. Briefly, two 20-base pair (bp) targeting sequences (TATAGGAGGCCTCTAGGCGC and CTGTTGGTGCACGTCGCCGA) were introduced separately into the px458 plasmid (Addgene). HeLa and Jurkat cells were co-transfected with both plasmids. After 72 h, single clones were selected for expression of GFP by fluorescence-activated cell sorting (FACS) and seeded on a 96-well plate. After 12 days, single clones were analyzed by immunoblotting to confirm the absence of ATG9A.

### Virus production

VSV-G-pseudotyped virus stocks were prepared in HEK-293T cells by transfection with the indicated plasmids using Lipofectamine 2000 (Invitrogen) according to the manufacturer’s instructions. Briefly, Lenti-X HEK-293T cells were co-transfected with psPAX2 (Addgene), pADV (Promega), pMD2G (Addgene) and Nef-expressing construct in pLex F67 mEmerald to generate VSV-G pseudotyped viruses. The supernatant containing viruses was collected 48 h after transfection, and viruses were concentrated using lentivirus precipitation solution (Alstem) according to the manufacturer’s protocol. For production of pNL4-3 viruses, HEK-293T cells were co-transfected with WT, pNL4-3 or pNL4-3ΔNef in the presence of VSV-G expression vector pHCMV-G. After overnight transfection, the medium was replaced with fresh DMEM. Viruses were collected 24 h later and subjected to reverse transcriptase (RT) activity assay, as previously described [86].

### Virus assembly and release

HeLa (parental or ATG9A-KO) cells were transfected with the indicated plasmids or infected with RT-normalized VSV-G-pseudotyped HIV-1 (NL4-3 or NL4-3ΔNef). Jurkat cells were infected with RT-normalized NL4-3 or NL4-3ΔNef particles pseudotyped with VSV-G. After 8 h of infection, cells were washed and medium replaced. Virus-containing supernatants were filtered 48 h later, and virus particles were collected by ultracentrifugation. A portion of virus-containing supernatant was stored for infectivity and RT assays. Cell and viral pellets were lysed [87]. The virus release efficiency (VRE) was calculated as the amount of virion-associated CAp24 as a fraction of total (cell- and virion-associated) CAp24 quantified by immunoblot analysis.

### Infectivity assay

For LUC-based, single-cycle infectivity assays, RT-normalized virus stocks were used to infect the CD4^+^/CXCR4^+^/CCR5^+^ HeLa derivative TZM-bl (obtained from J. Kappes through the National Institutes of Health AIDS Research and Reference Reagent Program, Bethesda, MD). This indicator cell line contains integrated copies of the β-galactosidase and LUC genes under the control of the HIV-1 LTR [88]. Infection efficiency was determined by measuring LUC activity 48 h post-infection as previously described [89].

### Statistical analysis

Analysis and plotting of data were performed using the GraphPad Prism 7.0 software (GraphPad Software, La Jolla California USA) and are expressed as the mean ± SD. The statistical significance of multiple samples was assessed via a one-way ANOVA analysis of variance (ANOVA), in addition to a Holm-Šídák post hoc test in some case. The unpaired *t* test was applied when two groups were compared. The results were accepted as significantly different when p ≤ 0.05, p ≤ 0.01, p ≤ 0.001 or p ≤ 0.0001.

## Additional files

**Additional file 1: Table S1.** Identification of HIV-1 and SIV Nef interactors by the TAP-MS protocol outlined in Fig. 1b. Initial datasets were filtered against the experimental control of the empty vector. The “IPI” column provides the International Protein Index identification code referring to the name of the protein in the “User input” column. The “unique peptide” column indicates the number of distinct peptides used to identify the protein whereas the “total peptide” column indicates the total number of identified peptides matched for the protein. Raw data were filtered against the CRAPome database and several values were generated. The experiment appeared (ExpAP) ratio indicates the number of times that protein appeared in a control experiment divided by the total number of experiments analyzed. The column “ExpAP calculated” provides the value of the calculated ratio from the “ExpAP” column. The average spectral count (Ave SC) is the average number of peptides in the experiments where the protein appeared. Max SC is the highest number of peptides found in a single experiment. By multiplying AveSC and ExpAP, we obtained an estimate of both the number of times the protein was identified in a negative control and its abundance when it was identified.

**Additional file 2: Fig. S1.** HIV and SIV Nef interactors. (A) BioGRID interaction map of SIV Nefs with host proteins identified by TAP-MS. (B) BioGRID interaction map of proteins identified by TAP-MS that interact with at least two Nef proteins. **Fig. S2.** Nef has no effect on the punctate appearance and staining intensity of endogenous LC3-II. (A) WT HeLa cells, stably expressing HIV-1 Nef-GFP or GFP, were fixed, permeabilized, immunostained with antibody to LC3 and imaged by confocal microscopy. Scale bar: 10 μm. (B) The same analysis was performed on Jurkat cells. **Fig. S3.** Nef has little or no effect on levels of autophagy proteins. Lysates of WT Jurkat and HeLa cells, stably expressing GFP (−) or HIV-1 Nef-GFP (+), were analyzed by SDS-PAGE and immunoblotting with antibodies to different autophagy proteins (beclin 1, ATG7, ATG5, ATG12, ATG16, LAMP-1 and SNAP29) and to actin. The positions of molecular mass markers (in kDa) are indicated on the left. The expression levels of the autophagy proteins were quantified relative to actin levels. Bar graphs represent the mean ± SD from four independent experiments. Statistical significance was evaluated using an unpaired Student’s t-test (ns: *p* > 0.05, *p < 0.05, **p < 0.01, ***p < 0.001). **Fig. S4.** Expression of ATG9A does not affect the levels or localization of Nef. WT and ATG9A-KO HeLa (A, B) or Jurkat (C, D) cells stably expressing Nef-GFP were analyzed by immunoblotting (A, C) and immunofluorescence microscopy (B, D) using antibodies to the indicated antigens. In A and C, the positions of molecular mass markers (in kDa) are indicated on the left. Bar graphs represent the mean ± SD of the levels of Nef-GFP relative to the levels of actin from three independent experiments. Statistical significance was determined using an unpaired Student’s t-test (ns: *p* > 0.05). In B and D, cells were fixed, permeabilized, immunostained with antibody to ATG9A and observed by confocal microscopy. Scale bars: 10 μm. **Fig. S5**. Localization of Nef is not affected by induction of autophagy. (A, B) WT HeLa cells, stably expressing HIV-1 Nef-GFP, were incubated in medium containing Torin1 for 2 h or HBSS medium for 1 h at 37 °C. Cells were then fixed, permeabilized, immunostained with antibody to ATG9A and LC3, and imaged by confocal microscopy. (A) Nef-GFP and ATG9A were imaged by confocal microscopy. Scale bar: 10 μm. (B) The number of LC3-II puncta per cell was counted for 20 cells per biological replicate for a total of three independent experiments using the ‘Analyze particles’ function of the Image J software. Bar graphs represent the mean ± SD of LC3-II puncta. Statistical significance was determined using a one-way ANOVA test (***p < 0.001, ****p < 0.0001). (C, D) The same analysis was performed on Jurkat cells. (**p < 0.01, ***p < 0.001). **Fig. S6.** ATG9A-Nef interaction requires residues in the 58–75 region of Nef. HeLa cells were transiently transfected with plasmids encoding WT HIV-1 Nef-FTS, Nef-FTS mutants (G2A, M20A, WL, EEEE or PxxP) (see Fig. 4a) or myrlysin-FTS, and cell extracts were incubated with Strep-Tactin beads. The isolated proteins were subjected to SDS-PAGE and immunoblotting with antibodies to FLAG and ATG9A. The positions of molecular mass markers (in kDa) are indicated on the left. The amount of ATG9A in the isolated samples was quantified relative to the amount of ATG9A in the input. Values were normalized to the ATG9A PD/input ratio in the WT condition. Bar graphs represent the mean ± SD from four independent experiments. Statistical significance was analyzed using an unpaired Student’s t-test (**p < 0.01, ***p < 0.001, ****p < 0.0001).

## Data Availability

All data generated or analyzed during this study are included in this published article and additional files.

## Abbreviations

ΔNef: Nef-deleted
ATG9A: autophagy-related 9A protein
AveSC: average spectral count
CAp24: HIV-1 capsid protein
cpz: chimpanzee
CRAPome: Contaminant Repository for Affinity Purification Mass Spectrometry Data
DSP: dithiobis succinimidyl propionate
Env: envelope glycoprotein
ExpAP: experiment appeared ratio
FTS: one FLAG followed by two strep tags
HIV: human immunodeficiency virus
IB: immunoblotting
KO: knockout
LTR: long terminal repeat
LUC: luciferase
mac: macaque
MS: mass spectrometry
PD: pull-down
RT: reverse transcriptase
SIV: simian immunodeficiency virus
smm: sooty mangabey
TAP: tandem affinity purification
TGN: *trans*-Golgi network
VSV-G: vesicular stomatitis virus G glycoprotein
WT: wild-type
