# Supplementary material for: The autophagy protein ATG9A promotes HIV-1 infectivity
Source: Retrovirology. 2019 Jul 3;16:18. doi: 10.1186/s12977-019-0480-3 (PMC6607583; doi:10.1186/s12977-019-0480-3)

Supplementary figure 1

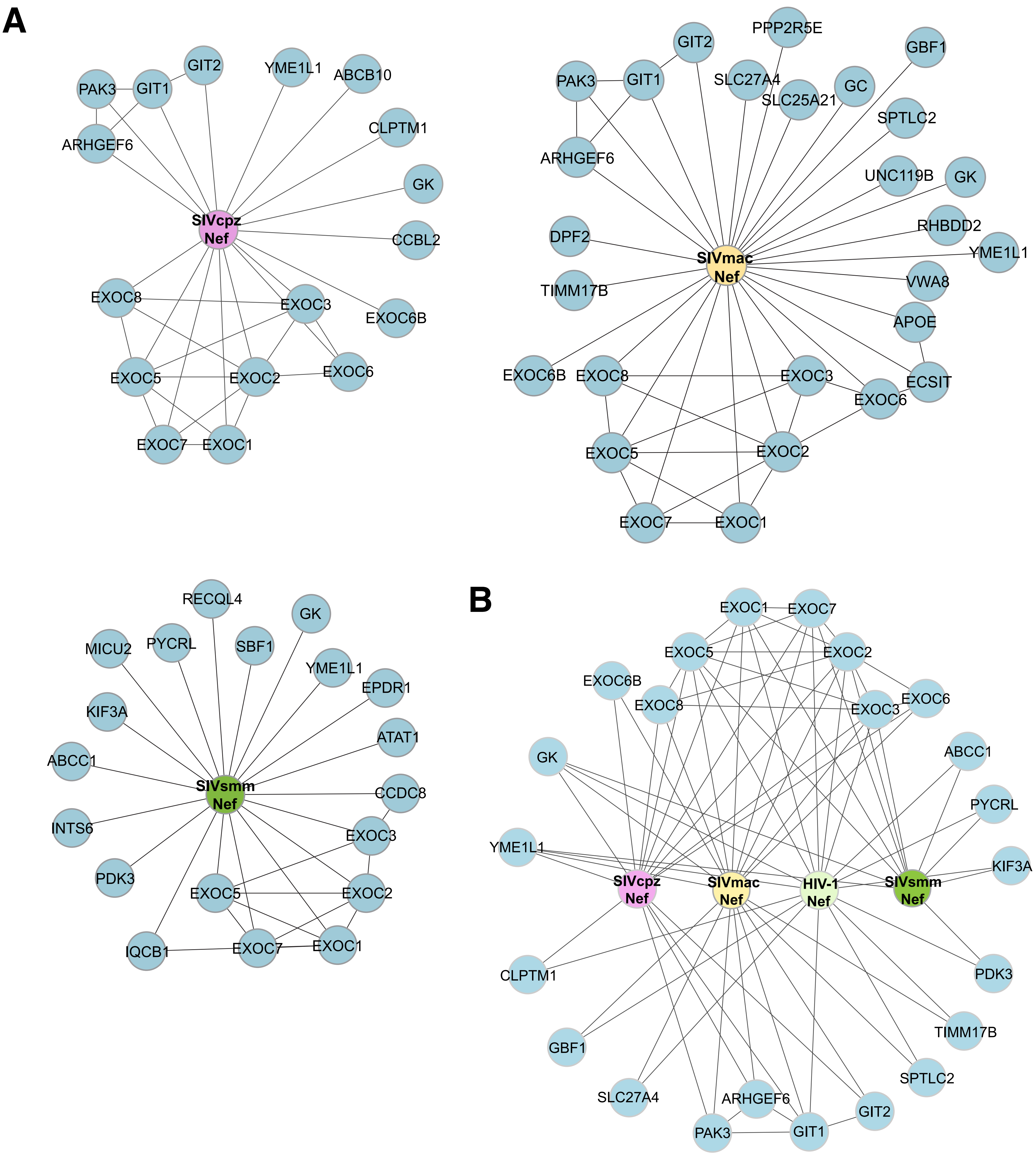

Supplementary figure 2

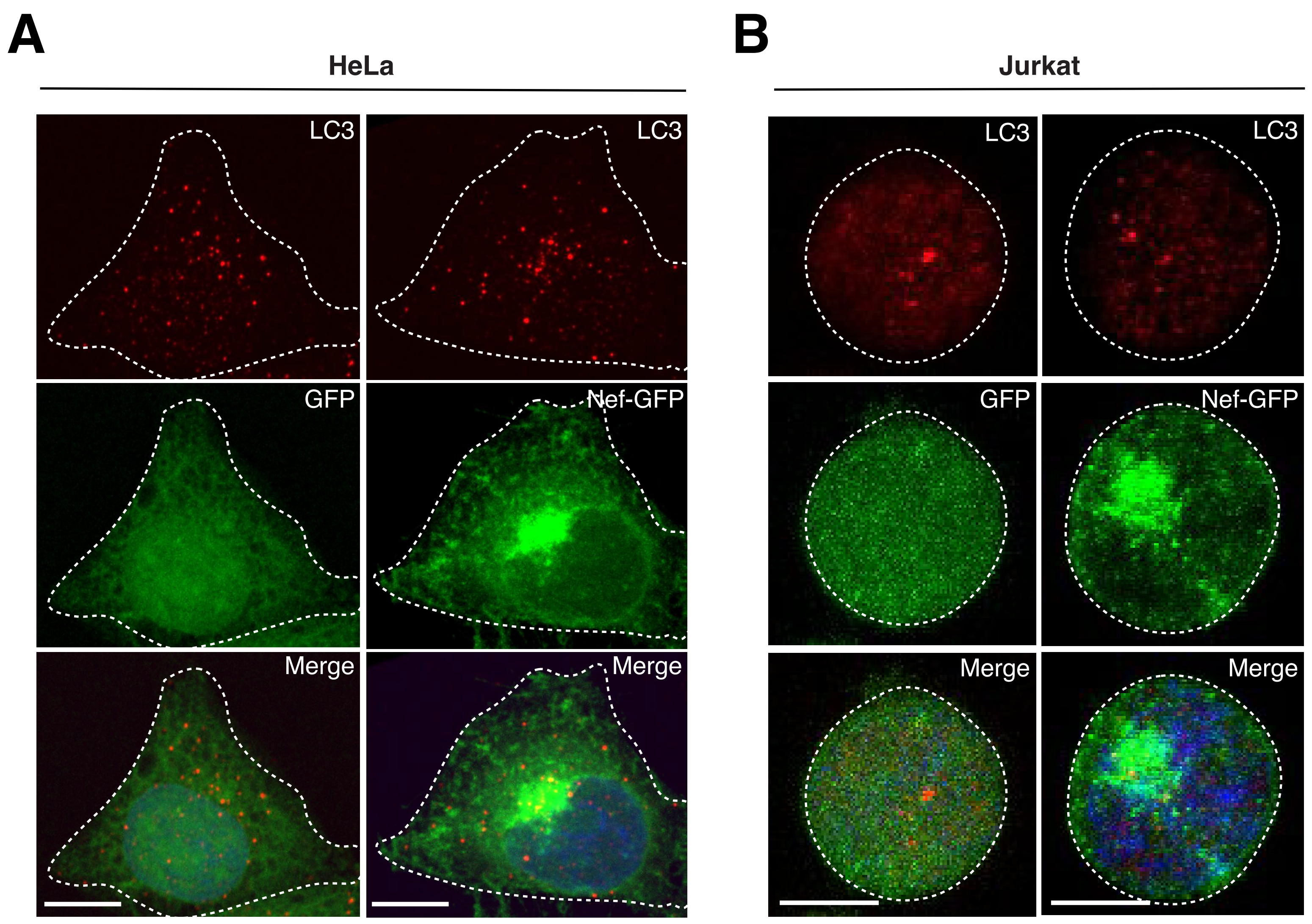

Supplementary figure 3

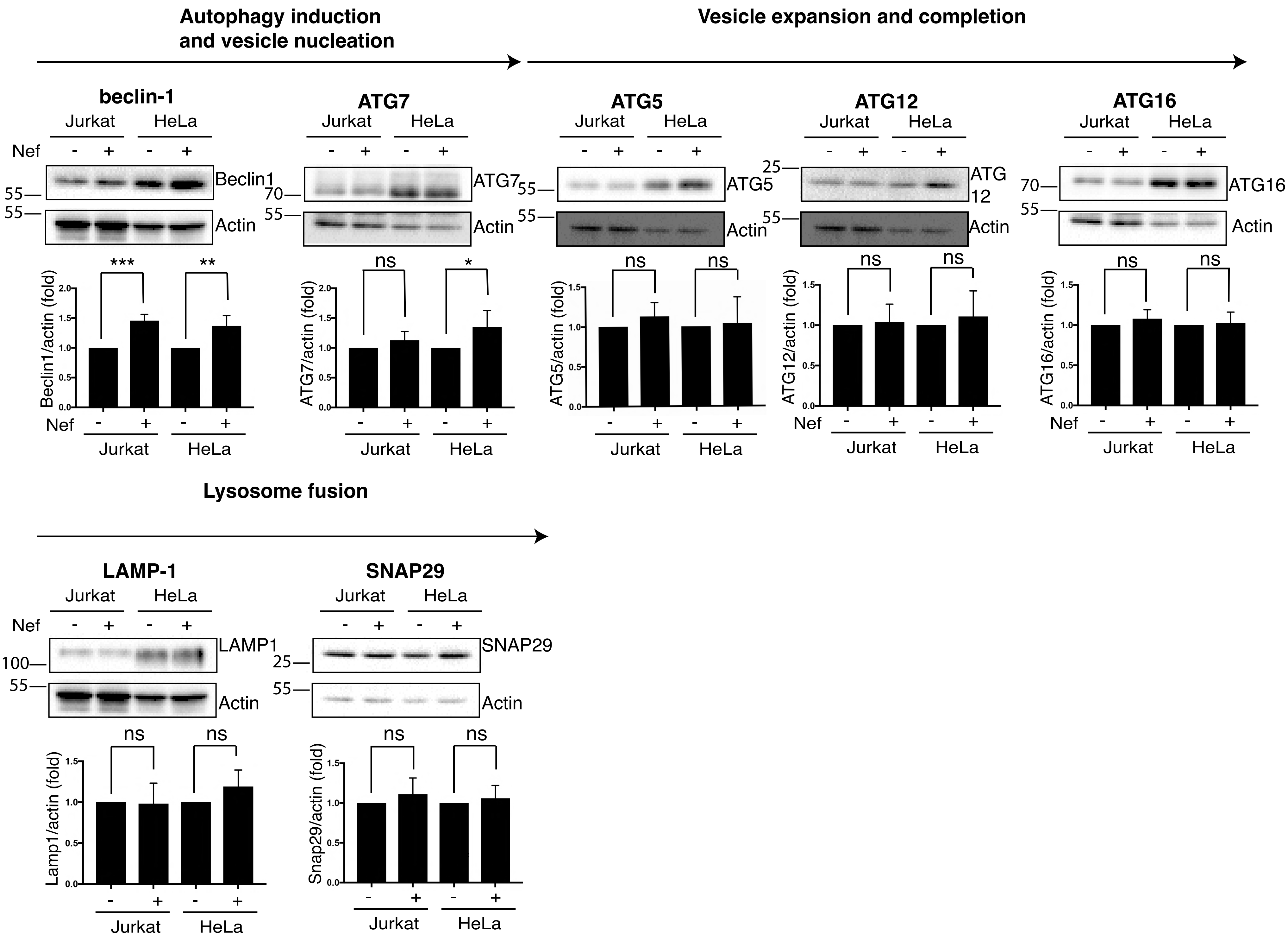

Supplementary figure 4

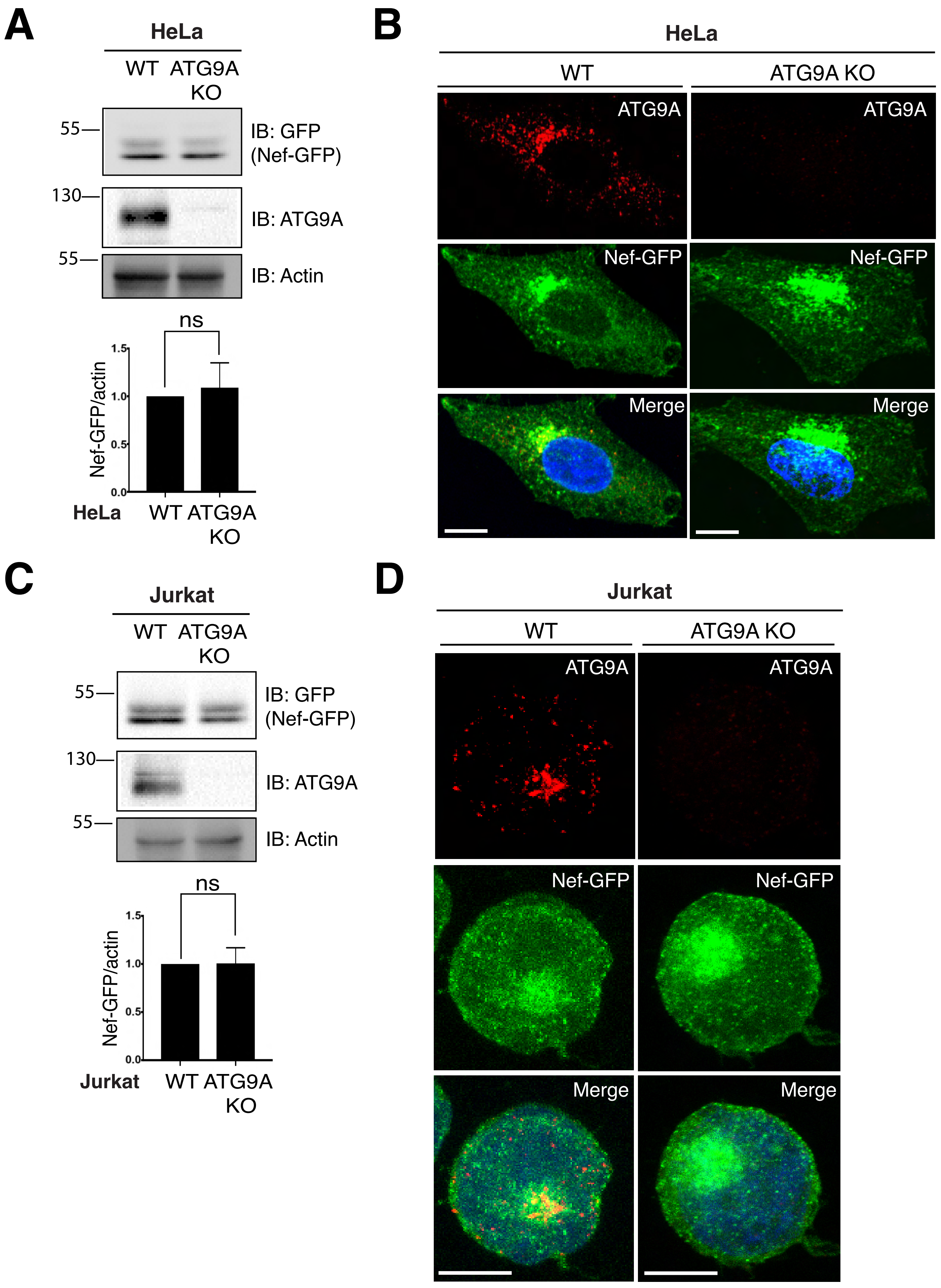

Supplementary figure 5

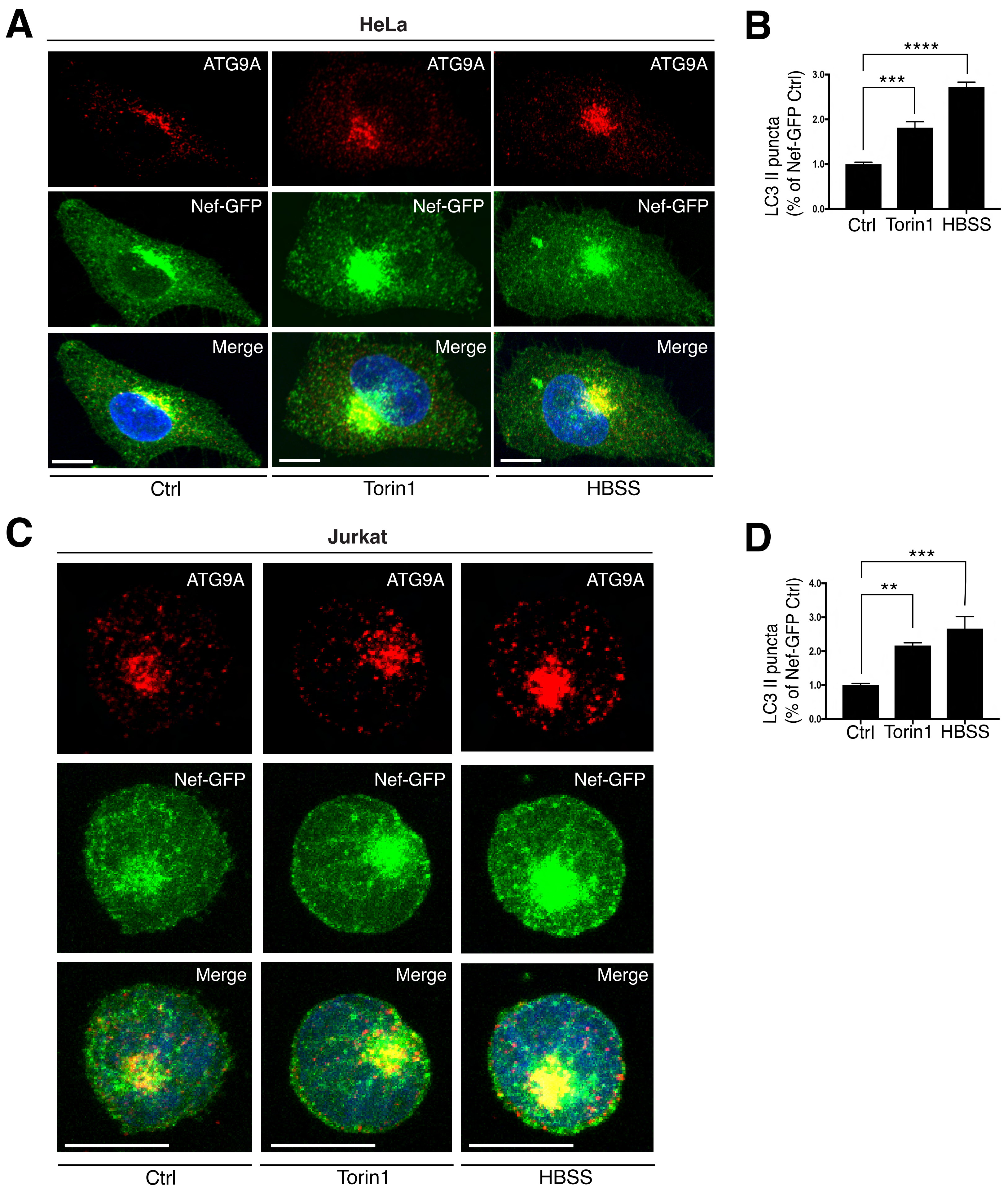

Supplementary figure 6

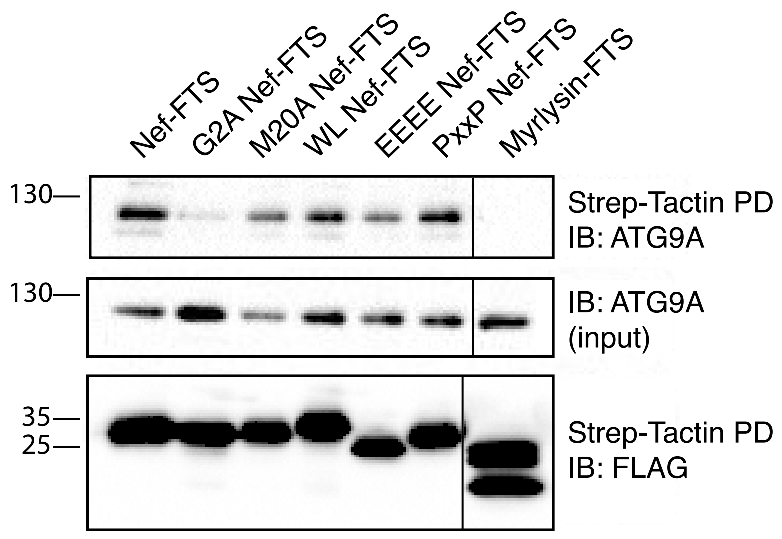

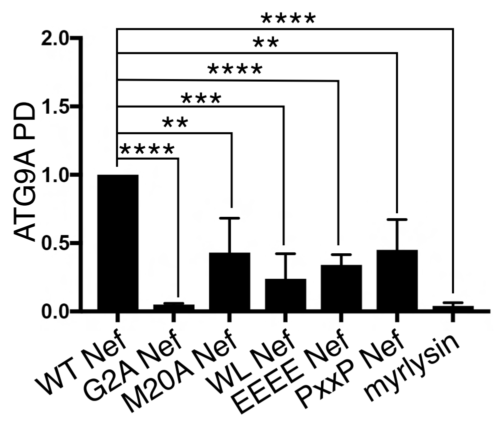

Supplement: Supplementary file 2 — Additional file 2: Fig. S1. HIV and SIV Nef interactors. (A) BioGRID interaction map of SIV Nefs with host proteins identified by TAP-MS. (B) BioGRID interaction map of proteins identified by TAP-MS that interact with at least two Nef proteins. Fig. S2. Nef has no effect on the punctate appearance and staining intensity of endogenous LC3-II. (A) WT HeLa cells, stably expressing HIV-1 Nef-GFP or GFP, were fixed, permeabilized, immunostained with antibody to LC3 and imaged by confocal microscopy. Scale bar: 10 μm. (B) The same analysis was performed on Jurkat cells. Fig. S3. Nef has little or no effect on levels of autophagy proteins. Lysates of WT Jurkat and HeLa cells, stably expressing GFP (−) or HIV-1 Nef-GFP (+), were analyzed by SDS-PAGE and immunoblotting with antibodies to different autophagy proteins (beclin 1, ATG7, ATG5, ATG12, ATG16, LAMP-1 and SNAP29) and to actin. The positions of molecular mass markers (in kDa) are indicated on the left. The expression levels of the autophagy proteins were quantified relative to actin levels. Bar graphs represent the mean ± SD from four independent experiments. Statistical significance was evaluated using an unpaired Student’s t-test (ns: p > 0.05, *p < 0.05, **p < 0.01, ***p < 0.001). Fig. S4. Expression of ATG9A does not affect the levels or localization of Nef. WT and ATG9A-KO HeLa (A, B) or Jurkat (C, D) cells stably expressing Nef-GFP were analyzed by immunoblotting (A, C) and immunofluorescence microscopy (B, D) using antibodies to the indicated antigens. In A and C, the positions of molecular mass markers (in kDa) are indicated on the left. Bar graphs represent the mean ± SD of the levels of Nef-GFP relative to the levels of actin from three independent experiments. Statistical significance was determined using an unpaired Student’s t-test (ns: p > 0.05). In B and D, cells were fixed, permeabilized, immunostained with antibody to ATG9A and observed by confocal microscopy. Scale bars: 10 μm. Fig. S5. Localization [file 12977_2019_480_MOESM2_ESM.pdf]
